# Multi-omics analysis and experiments uncover the link between cancer intrinsic drivers, stemness, and immunotherapy in ovarian cancer with validation in a pan-cancer census

**DOI:** 10.3389/fimmu.2025.1549656

**Published:** 2025-05-08

**Authors:** Yilin Li, Cen Chen, Xiaoyu Ji, Ningxiao Jiang, Fei Wang, Xiangqian Gao, Weiwei Chen, Qiang Tang, Yan Li, Shinan Zhang, Gaofeng Qin, Yingjiang Xu, Yanlin Wang, Lingwen Kong, Lei Han, Jie Mei

**Affiliations:** ^1^ Department of Obstetrics and Gynecology, Sichuan Provincial People's Hospital, School of Medicine, University of Electronic Science and Technology of China, Chengdu, Sichuan, China; ^2^ Department of Oncology, Huashan Hospital, Fudan University, Shanghai, China; ^3^ Department of Reproductive Medicine, Binzhou Medical University Hospital, Binzhou Medical University, Binzhou, Shandong, China; ^4^ Medical Research Center, Binzhou Medical University Hospital, Binzhou Medical University, Binzhou, Shandong, China; ^5^ Department of Pathology, Binzhou Medical University Hospital, Binzhou Medical University, Binzhou, Shandong, China; ^6^ Meishan City People’s Hospital, Meishan, Sichuan, China; ^7^ Department of Traditional Chinese Medicine, Binzhou Medical University Hospital, Binzhou, Shandong, China; ^8^ Department of Interventional Vascular Surgery, Binzhou Medical University Hospital, Binzhou, Shandong, China; ^9^ Institute of Intergrated Traditional Chinese and Western Medicine, Huashan Hospital Fudan University, Shanghai, China

**Keywords:** cancer stemness, intrinsic heterogeneity, immunotherapy therapy, CSE1L, ovarian cancer

## Abstract

**Background:**

Although immune checkpoint inhibitors (ICIs) represent a substantial breakthrough in cancer treatment, it is crucial to acknowledge that their efficacy is limited to a subset of patients. The heterogeneity and stemness of cancer render its response to immunotherapy variable, warranting the identification of robust biomarkers for evaluation.

**Methods:**

Publicly available Ovarian Cancer (OV) single-cell RNA (scRNA) sequence dataset was collected and analyzed to elucidate the intrinsic driver gene of OV cancer cells. Through genome-scale CRISPR screening of RNA sequencing data from Project Achilles, essential genes specific to OV were identified. A novel cancer stem cell index (CSCI) was developed and validated using multiple advanced algorithms and large-scale datasets, as well as corresponding clinical features, including 14 OV transcriptomic datasets, 7 pan-cancer ICI transcriptomic cohorts and one melanoma scRNA dataset derived from PD-1 treated patients.

**Results:**

Chromosomal 20q gain, 8q gain, and 5q loss have been identified as ovarian cancer-specific driving variations. By analyzing large-scale datasets of ovarian cancer transcriptomics, including scRNA and CRISPR cell line datasets, we have identified a gene set that influences tumor intrinsic drivers and stemness properties. We then developed the CSCI to predict the prognosis and response to immunotherapy in ovarian cancer patients using advanced machine learning algorithms. When applied to PD1/PD-L1 ICI transcriptomic cohorts, CSCI consistently and accurately predicts tumor progression and immunotherapy benefits, with a mean AUC greater than 0.8. Notably, compared to previously established signatures, CSCI demonstrates better predictive performance across multiple ovarian cancer datasets. Intriguingly, we discovered that amplification of CSE1L enhances the stemness of tumor-initiating cells, facilitates angiogenesis, and the formation of ovarian cancer, which can serve as a potential therapeutic target. Finally, experiments validated that CSE1L promotes progression, migration, and proliferation of ovarian cancer.

**Conclusions:**

Our study has uncovered a robust correlation between variations in cancer intrinsic drivers and stemness, as well as resistance to immunotherapy. This finding provides valuable insights for potential strategies to overcome immune resistance by targeting genes associated with stemness.

## Introduction

The exceptional development in immuno-oncology and the emergence of immunotherapeutic medications like ICIs and chimeric antigen receptor (CAR) T cell therapy, as of the present, furnish promising strategies to stimulate the inherent immune system for conquering cancers (1). However, a significant proportion of patients fail to experience substantial benefits from immunotherapy. Consequently, there is an urgent need to identify eligible patient populations for effective immunotherapy.

The effectiveness of immunotherapy relies on a complex network involving multiple modulators. Conventional biomarker inquiries have chiefly concentrated on analyzing the large quantity of data gotten from RNA sequencing (RNA-Seq) of uninjured tumor tissue or the tumor immune microenvironment (TME) (2). Possibly owing to the tumor genetic diversity, the TME displays significant differences among various cancer varieties and even between distinct individuals (3). On top of that, previous studies have proposed certain biomarkers associated with immune response, such as tumor mutation burden (TMB), which plays a crucial role in initiating the cancer-immunity cycle (4). Nevertheless, because of tumor genetic heterogeneity, these biomarkers fail to fully stratify patients for optimal therapeutic benefits. As an instance, The often-used TMB may fail to predict the potency of combined anti-Programmed Cell Death 1 (anti-PD-1) and anti-Cytotoxic T-Lymphocyte Associated Protein 4 (anti-CTLA-4) treatment in a number of cancer varieties (4), underscoring the necessity to establish robust markers and optimize biomarker combinations. Advancing the detection of novel triggers specific to cancer cells, the appearance of single-cell RNA sequencing (scRNA-Seq) currently allows for the examination of genomic characteristics and expression fashions at the single-cell level (5).

Cancer stem cells (CSCs), which are involved in the commencement, development, and dissemination of tumors, are cells possessing the capacity for self-renewal (6). n the realm of cancer, increasing evidence shows a substantial correlation between stemness characteristics and immune avoidance as well as resistance (7). Within 21 varieties of solid cancers, a prior study disclosed an inverse correlation between heightened stemness and the penetration of immune cells (8). Within pan-cancer cohorts, Zhang Z discovered a reverse correlation between the stemness of tumors and the effectiveness of ICI treatment (9). Nevertheless, the relationship between tumor stemness and ICI efficacy in ovarian cancer has been disregarded. Leveraging the powerful gene expression-based stemness index (mRNAsi) developed by Lian H et al., To examine the effect of stemness on ICI, we accurately depicted cancer stemness and pinpointed stemness-associated genes in large-scale ovarian cancer groups (9).

We uncovered the innate distinctions particular to ovarian cancer and detected a reverse correlation between cancer-intrinsic variation, cancer stemness, and the outcomes of ICI treatment in SKCM single cell ICI cohorts. In this investigation, via carrying out integrated analyses regarding the ovarian cancer transcriptome, single-cell, and CRISPR cell line datasets (10). Subsequently, we developed a cancer stemness cell index (CSCI) through integrative analysis of 15 ovarian cancer cohorts comprising 2518 patients. The precision of CSCI in predicting immunotherapy response was further evaluated and validated using 7 independent anti-PD-1 ICI cohorts and the submap algorithm. Intriguingly, we identified two stemness-associated genes, RAD21 and CSE1L, driven by intrinsic tumor variations. Even though RAD21 has been previously noted for its copy number amplification helping with tumor immune avoidance and lessening the efficacy of immunotherapy (11), we also found that CSE1L amplification can facilitate tumor cell invasion through the activation of JAK-STAT and VEGF signaling pathways. This discovery suggests that CSE1L may represent a novel potential immunotherapeutic target in the future. Overall, our comprehensive analysis provides valuable insights into the involvement of intrinsic variations and stemness in ovarian cancer immunotherapy.

## Methods

### Cell lines

Human ovarian cancer cell lines SK-OV-3 and A2780 were supplied by the Cell Bank of the Committee for Conservation of Typical Cultures of the Chinese Academy of Sciences.These cell lines were maintained using Dulbecco’s Modified Eagle Medium (DMEM) sourced from Gibco (New York, USA), enriched with 10% fetal bovine serum.The medium was supplemented with penicillin and streptomycin at a concentration of 100 IU/mL (Gibco, New York, USA).

### Immunohistochemistry

Ovarian cancer tissues and para-carcinoma tissues removed during operations were rinsed with sterile PBS to remove blood stains, and then fixed in paraformaldehyde solution for 24 hours. After the tissue samples were extracted and encased in paraffin, they were consecutively sliced to a thickness of 4 μm. Sections were affixed to slides using the floating patch method, placed in an oven at 60°C, and baked overnight. They were then deparaffinized twice in xylene solution and rinsed in a gradient ethanol solution after soaking and rinsing in PBS. The sections were placed in citrate antigen retrieval solution and incubated under high pressure for 1 hour. The sealing solution was carefully aspirated, diluted primary antibody was added dropwise, and the sections were placed in a wet box for overnight incubation at 4°C. Then the wet box was rewarmed at room temperature for 1 hour. The application of the secondary antibody was carried out by dropping. After that, the sections were incubated in the wet box for 2 hours, rinsed in PBS five times, and the excess PBS around the samples was wiped off. Add DAB chromogen solution, observe the degree of color development under the microscope, and rinse with water to terminate the reaction when the appropriate color depth is reached. The slices were washed twice in deionized water, stained with hematoxylin and eosin, rinsed under running water until colorless, then dehydrated in a gradient ethanol solution, immersed in xylene twice, each time for 5 minutes, sealed with neutral gum, and dried in a fume hood. Finally, the samples were examined using a light microscope. The experimental procedures, protocols and informed consent for this study were all approved by the Research Ethics Committee of Binzhou Medical University Hospital. We confirm that researches involving human participants including collection of histological specimens and immunohistochemical staining of patients were all performed in accordance with the Declaration of Helsinki. From all the individuals involved and/or their legal protectors, the consent after thorough information communication was obtained.

### Knockdown in ovarian cancer cell lines

The siRNA targeting CSE1L was synthesized by Biotend Co., Ltd. Transfections with 50 nM of this siRNA were carried out for 24 hours using the Lipofectamine 3000 transfection kit (Thermo Fisher Scientific, Waltham, Massachusetts, USA).

### Western blotting

For the Western blot analysis, the cultured cells were first rinsed with ice-cold PBS. Total cell protein lysates were then extracted at 4°C using RIPA lysis buffer (Beyotime, Shanghai, China) supplemented with 1% protease inhibitor cocktail (MedChemExpress, New Jersey, USA). After centrifugation at 12,000 g for 20 minutes at 4°C, the supernatant containing proteins was collected and mixed with loading buffer. The samples were subsequently separated by 10% SDS-PAGE and transferred onto a PVDF membrane. In the environment of room temperature, the membrane was covered with 5% skim milk for two hours, followed by an overnight incubation at 4°C with primary antibodies. Following washes with Tris Buffered Saline, the membrane was incubated with secondary antibodies, and protein bands were detected using enhanced chemiluminescence reagents (Beyotime, Shanghai, China). The primary antibodies used in this analysis included CSE1L (22219-1-AP, Proteintech, Wuhan, China) and Alpha Tubulin (11224-1-AP, Proteintech, Wuhan, China).

### Cell viability measurement

To assess cell viability, 2×10^3 cells were plated in each well of a 96-well plate and incubated for a predetermined period. Following this, 10 μl of CCK-8 reagent (Dojindo Molecular Technologies, Kumamoto, Japan) was added to each well and incubated for one hour. The measurement of absorbance at 450 nm (OD450) was subsequently carried out for evaluation.

### Assessment of cell migration abilities

To assess cell migration capability, adjust the cell density for seeding to achieve approximately 100% confluence before making a scratch. Use the tip of a 10 μl pipette to create a linear wound on the cell surface. Add serum-free medium and incubate at 37 °C with 5% CO_2_. Capture images using an inverted microscope at 0 hours and 24 hours post-incubation for quantitative analysis.

Simultaneously, 4×10^4 cells were suspended in 200 μL of culture medium and seeded into the upper chamber of Transwell plates (BD Biosciences, Bedford, MA, USA). Meanwhile, 600 L of medium with 10% FBS was introduced into the bottom compartment. After a 24-hour incubation at 37 °C, the cells on the outside of the Transwell membrane were fixed with 4% paraformaldehyde for 30 minutes and then stained with 0.25% crystal violet for an additional 30 minutes. Once the cells inside the chamber were removed, the migrated cells on the outside of the membrane were photographed and quantified.

### Retrieval and preprocessing of large-scale ovarian cancer datasets

The ovarian cancer RNA sequencing (RNA-seq) data and survival-related data sourced from The Cancer Genome Atlas (TCGA) were fetched from the UCSC Xena data portal (12). Gene Expression Omnibus (GEO): fourteen OV GEO cohorts with detailed survival data were downloaded, namely GSE13876, GSE138866, GSE140082, GSE14764, GSE17260, GSE18520, GSE18521, GSE19829, GSE26712, GSE30161, GSE31245, GSE49997, GSE63885 and GSE9891.

### Immunotherapy-associated datasets collection

Multiple datasets with anti-PD-L1/PD-1 cohort were collected in the study to investigate the association between immunotherapy efficacy and cancer stemness. The Rose TL cohort (13) (GSE176307: ICB treated metastatic urothelial cancer). The Jung H cohort (14) (GSE135222: anti-PD-1/PD-L1 treated non-small cell lung carcinoma) and the Riaz N cohort (15) (GSE91061: anti-CTLA4 and PD-1 treated melanoma) were obtained from GEO. The Liu/VanAllen cohort (16) (phs000452.v3: anti-PD1/CTLA4-treated metastatic melanoma) was downloaded from dbGaP database (https://www.ncbi.nlm.nih.gov/gap/).The Necchi cohort (17) (IMvigor210: Atezolizumab treated advanced or metastatic urothelial carcinoma) was downloaded using “IMvigor210CoreBiologies” R package. The Wang GY cohort (18) (anti-PD-1/PD-L1 treated melanoma) was downloaded from the **Supplementary Tables** of this research. The Braun DA cohort (19) (anti-PD-1 treated advanced clear cell renal cell carcinoma) was downloaded from the **Supplementary Tables** of this research. ICI-treated OV cohort was downloaded from cds database (http://cdsdb.ncpsb.org.cn/) which also can be obtained from GEO database (GSE188249). In addition, Gene expression and clinical information of these datasets with immunotherapy treated were collected. All cohorts used in this article were described in **Supplementary Table S1**.

### Single cell datasets of OV and ICI treated SKCM collection

Preprocessed gene expression profiles of ovarian cancer (OV) were obtained from the GEO database under accession number GSE184880 (20), comprising five non-malignant tissues and seven high-grade serous ovarian cancer tissues. Additionally, with the aim of exploring the correlation between the stemness of cancer cells and the efficacy of immunotherapy, a melanoma cohort that incorporated both data on ICI response and single-cell RNA sequencing was accessed through the GEO accession number GSE115978 (10).

### Identifying essential OV genes

The genome-wide CRISPR screening of OV cells was obtained from the DepMap portal (https://depmap.org/portal/download/). The CERES algorithm (21) was used to calculate dependency scores for approximately 17,000 candidate genes. Essential genes were identified as those with a CERES score of <−1 in 75% of OV cell lines (n = 73).

### Identifying cancer intrinsic heterogeneity and stemness related genes and construction of CSC prediction model

**Figure 1:**
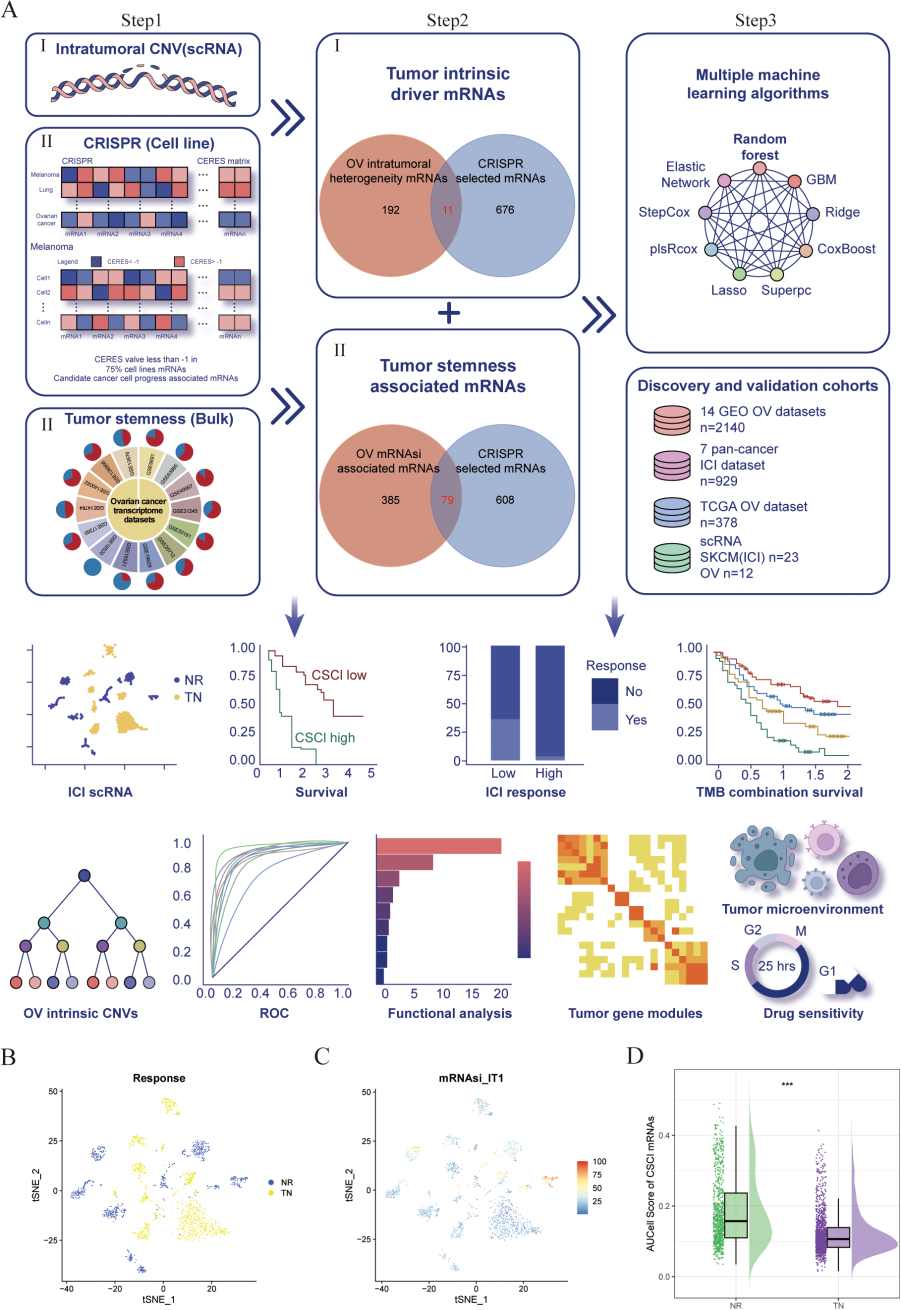
Identification and validation of a negative association between cancer stemness and ICI response in cancer. **(A)** Workflow of identification of Cancer intrinsic heterogeneity and stemness associated mRNAs and construction of predictors via multiple machine learning algorithms. **(B–D)** t-Distributed Stochastic Neighbor Embedding (tSNE) plot of malignant cells from SKCM. **(B)** tSNE plots label the malignant cells by response phenotype. **(C)** tSNE plot of AUCell score of CSC associated genesets in malignant cells, red indicates higher scores (high stemness) while blue indicates lower scores (low stemness). **(D)** Raincloud plot of AUCell scores by response phenotype (NR vs. TN) in SKCM cohort. The center of the box plot are median values, the bounds of the box are 25% and 75% quantiles (Wilcoxon test; ***P < 0.001). NR, non-responders; TN, treatment naïve patients.

We constructed an innovative framework for cancer intrinsic heterogeneity and stemness prediction modeling, illustrated in **Figure 1A**. Initially, to elucidate genes driving tumor intrinsic heterogeneity, we analyzed the OV single-cell dataset (GSE115978) with inferCNV (22) R software. Our analysis revealed widespread chromosomal alterations across OV tumor cells, specifically chr 5q deletions and 8q/20q amplifications (**Figure 1A**: step1-I; S1F-H). Given that genomic amplifications potentially enhance gene expression and tumorigenic properties, we focused on 203 heterogeneity-associated genes located within amplified regions on chromosomes 8q and 20q. Subsequently, to pinpoint essential genes in OV pathogenesis, we examined genome-wide CRISPR-based functional screens from DepMap. This investigation identified 687 genes essential for maintaining viability across 73 OV cell lines (**Figure 1A**: step1-II). By intersecting these datasets, we pinpointed 11 critical tumor-intrinsic genes (**Figure 1A**: step2-I).

Concurrently, recognizing stemness contributions to immunotherapy resistance, we compiled 14 OV transcriptome datasets from GEO (**Figure 1A**: step1-III, **Supplementary Table S1**). We employed the mRNA-based stemness index (mRNAsi) to quantify stemness characteristics within each sample, following methodologies described in previous studies (23). Our approach utilized reference stem cell gene signatures with weighted coefficients (23) to calculate stemness scores through Spearman correlation analysis between sample expression profiles and canonical stem cell patterns. These correlations were normalized to a 0-1 scale for comparative assessment, with higher values indicating enhanced stem-like properties. We then examined relationships between global mRNA expression and stemness indices, defining mRNAsi-associated transcripts as those showing significant positive correlations (Cor > 0, P < 0.05) in at least 50% of datasets (7/14), yielding 464 stemness-linked mRNAs (**Figure 1A**: step1-III). Through dataset integration, we established 79 stemness-related genes fundamental to tumor cell viability (**Figure 1A**: step2-II; **Supplementary Table S2**).

Finally, using seven OV cohorts with survival information, we developed our CSC prediction model. We implemented multiple machine learning approaches: random forest (RSF), elastic network (Enet), gradient boosting machine (GBM), ridge regression, Stepcox, plsRcox, CoxBoost, LASSO, and SuperPC. By inputting the expression matrix that integrates cancer stemness with intrinsic driver genes, we employed multiple machine learning algorithms to forecast both the survival duration and status of patients. Ultimately, scores for each patients were computed (**Figure 1A**: step 3). The C-index was determined using the scores predicted by the model, applying the ‘coxph’ function to assess the survival duration and status of patients. The model that achieved the highest average C-index across several datasets is deemed the most effective. In this process, survival, randomForestSRC, glmnet, plsRcox, superpc, gbm, CoxBoost, survivalsvm, BART, miscTools, and compareC R packages were involved.

### Gene set variation analysis and pathways enrichment analysis

To investigate the correlation between cancer immunity cycle and CSE1L, we employed the “GSVA” R package (24) for the execution of GSVA enrichment analysis.

### Immune cell infiltration abundance calculation

The matrices for estimating the infiltration of immune cells in patients with OV, which were calculated using the algorithms from the Tumor Immune Estimation Resource (TIMER) (25), were acquired by uploading the expression matrix and downloading them from the TIMER database (https://cistrome.shinyapps.io/timer/).

### Prediction of immunotherapy outcomes

To assess the efficacy of PD-1/CTLA4 immunotherapy, we began by calculating tumor immune dysfunction and exclusion (TIDE) scores using expression data from patients with thyroid cancer. Firstly, we performed a two-step normalization process on the gene expression matrix by first centering each sample by its median expression value and then centering each gene by its median expression across all samples, effectively removing systematic biases to make both samples and genes more comparable for downstream analysis. Subsequently, we processed these expression profiles on the TIDE (26) database platform (http://tide.dfci.harvard.edu/) to forecast patient outcomes by uploading the expression matrix to the website, where we selected ‘other’ in the ‘cancer type’ section and ‘no’ in the ‘Previous immunotherapy’ section. We created input files necessary for SubMap analysis through a careful data processing procedure. We commenced with the original raw expression data, imposing strict filters to eliminate genes with low expression (specifically, those exhibiting values below 1 in more than 90% of samples). To ensure the gene expression data was standardized, we identified overlapping gene lists across various datasets and conducted a log2 transformation by applying the formula (log2(x + 1)) to normalize the expression levels and address possible numerical difficulties. Sample classification involved dividing samples into two separate groups: low-CSCI and high-CSCI. Each group was assigned a numerical rank to aid in computational analysis, designating CSCI-low as rank 1 and CSCI-high as rank 2. The R function generateInputFileForSubMap() was employed systematically to create two essential files needed for SubMap analysis: SubMap.gct and SubMap.cls. The SubMap.gct file contains the log2-transformed gene expression matrix, maintaining the molecular specifics of each sample, while the SubMap.cls file provides the corresponding sample classification data along with their assigned numerical ranks. This methodology ensures that the generated files fulfill the rigorous input criteria of the SubMap algorithm, allowing for accurate comparative analysis of gene expression profiles across various immunological clusters.

### Evaluation of pathway activity using PROGENy

In order to assess the function of various pathways in patients with ovarian cancer (OV), PROGENy (27) was employed as a tool for inferring pathway activity based on gene expression. This technique entails the utilization of key genes responsive to pathways, which were obtained from an extensive assortment of perturbation experiments that are publicly accessible.

### Prediction of favorable drugs in high and low CSE1L groups and drug sensitivity screening

Using the ‘oncoPredict’ R package and the calcPhenotype method, the prediction of drug sensitivity in cell lines was accomplished by analyzing gene expression profiles. In order to estimate the IC50 of drugs, a ridge regression model was constructed based on the gene expression profiles of cell lines obtained from GDSC using the pRRophetic algorithm (28).

### Differential expression, survival analysis and statistical analysis

The comparison of various characteristics between high- and low- CSE1L groups was conducted using the Wilcoxon test. The Chisq test was employed to analyze the variance in immunotherapy response between low- and high- CSCI groups. Pearson’s correlation coefficient was used to calculate the relationship between mRNA and mRNAsi. To investigate the association between CSCI, CSE1L, and survival, Kaplan-Meier survival analysis was performed, with the log-rank test used to determine the significance of observed differences. The prognostic and immunotherapy benefits of CSCI were assessed using time-dependent receiver operating characteristic (ROC) curves, utilizing the ‘pROC’ R package (29). Additionally, patients were categorized based on the optimal threshold determined by the ‘survminer’ R package. A significance threshold of P or adjusted P < 0. 05 served as the threshold to determine statistical significance.

## Results

### Cancer intrinsic heterogeneity and stemness is associated with ICI resistance

To investigate the clonal organization and cellular origins of OV malignant cells, we initially acquired a single cell RNA profile of OV carcinoma. This profile consisted of five normal ovarian tissues and seven OV tissues. Our first step was to filter out cells that expressed less than 200 genes and eliminate cells with over 20% expression of mitochondrial genes. Following quality control measures, we then classified the filtered cells into eight primary cell types using established biomarkers. These cell types included T cells, NK cells, Fibroblast, Myeloid, Epithelial, B cells, Plasmablast, and Endothelial (**Supplementary Figures S1A, B**). Subsequently, we employed the inferCNV algorithm to examine the copy number variations (CNV) and clonality of the OV malignant cells derived from epithelial cells (ECs). Out of the 3,396 epithelial cells obtained from OV tissues, our analysis revealed that 2,807 ECs displayed high CNV scores and were therefore considered to be malignant (**Supplementary Figure S1C**).

Then, the evolutionary tree of phylogenetics was implemented to visually represent the clonality and progression of malignant cells from OV (**Supplementary Figure S1D**). The presence of 20q and 8q amplifications, as well as loss of 5q, were universally observed in 100% of malignant OV cells (**Supplementary Figures S1D, E**). To further analyze and identify the intrinsic drivers responsible for these genetic alterations, UpSet plots were utilized, revealing a collective count of 203 genes that were shared among eight subclonal cell populations harboring 20q/8q amplifications and 5q loss in OV samples (**Supplementary Figures S1F, G**; **Supplementary Table S3**). Additionally, to identify key candidate genes implicated in OV malignancy, an extensive investigation of DepMap-derived CRISPR-based loss-of-function screens was conducted on a genome-wide scale. Through this approach, a total of 687 genes were found to be critical for the survival of 73 OV cell lines. After conducting a thorough analysis, 11 copy number variations driven genes were selected, as they displayed significant impacts on the progression of ovarian cancer cells (**Figure 1A**). Interestingly, it was observed that all 11 of these genes exhibited copy number amplification, thus pointing towards a potential role of 20q/8q gain in influencing the survival of tumor cells.

Additionally, taking into consideration the potential role of cancer stemness in the resistance against ICIs, we gathered a total of 14 transcriptome datasets on ovarian cancer (OV) from the GEO database (**Figure 1A**; **Supplementary Table S1**). From these datasets, we calculated the mRNA expression-based stemness index (mRNAsi) for each patient (9). Using the Pearson correlation coefficient, we identified mRNAs that displayed a significant correlation with mRNAsi across multiple samples (Cor>0 & P<0.05). Then, we discovered 464 mRNAs that were positively correlated with the stemness index in at least half of the datasets (7 out of 14) and were deemed as tumor stemness-associated mRNAs. Among these, we selected 79 mRNAs by overlapping the OV mRNAsi-associated mRNAs with the mRNAs selected by CRISPR (**Supplementary Table S3**). All these cancer stemness and intrinsic drivers were chosen for further analysis. We then employed AUCell enrichment analysis (30) to confirm the high specificity of the gene set comprising cancer stemness and intrinsic drivers in recognizing neoplastic cells in scRNA-seq data of OV (**Supplementary Figure S2A**). To verify the impact of cancer stemness and inherent driver genes on the efficacy of immunotherapy, we initially employed a formerly published scRNA-seq dataset of melanoma (SKCM) patients treated with ICI to analyze the correlation between cancer stemness and the outcomes of ICI treatment (10). After excluding patients without data on malignant cells, we included a total of 23 patients from this cohort, consisting of 10 non-responders (NR) and 13 treatment-naïve (TN) patients. Ideally, it would have been preferable to conduct a comparison of cancer stemness between individuals who responded to ICI treatment (R) and those who did not respond (NR). However, the available dataset did not include data specifically for responders. Since treatment-naïve patients are likely to include both potential responders and non-responders, we proceeded to compare the levels of stemness between the non-responder group (NR) and the treatment-naïve group (TN), as previously explained (10). As shown in **Figures 1B, C**, the NR subgroup exhibited a higher abundance of cancer cells with elevated stemness scores. Further analysis uncovered that tumors from the NR subgroup exhibited significantly elevated levels of stemness (P < 0.001, **Figure 1D**), suggesting a negative association between cancer intrinsic driver and stemness with immune checkpoint inhibitor outcomes.

### Construction of cancer stemness cell index based on large scale OV RNA-seq cohorts

**Figure 2:**
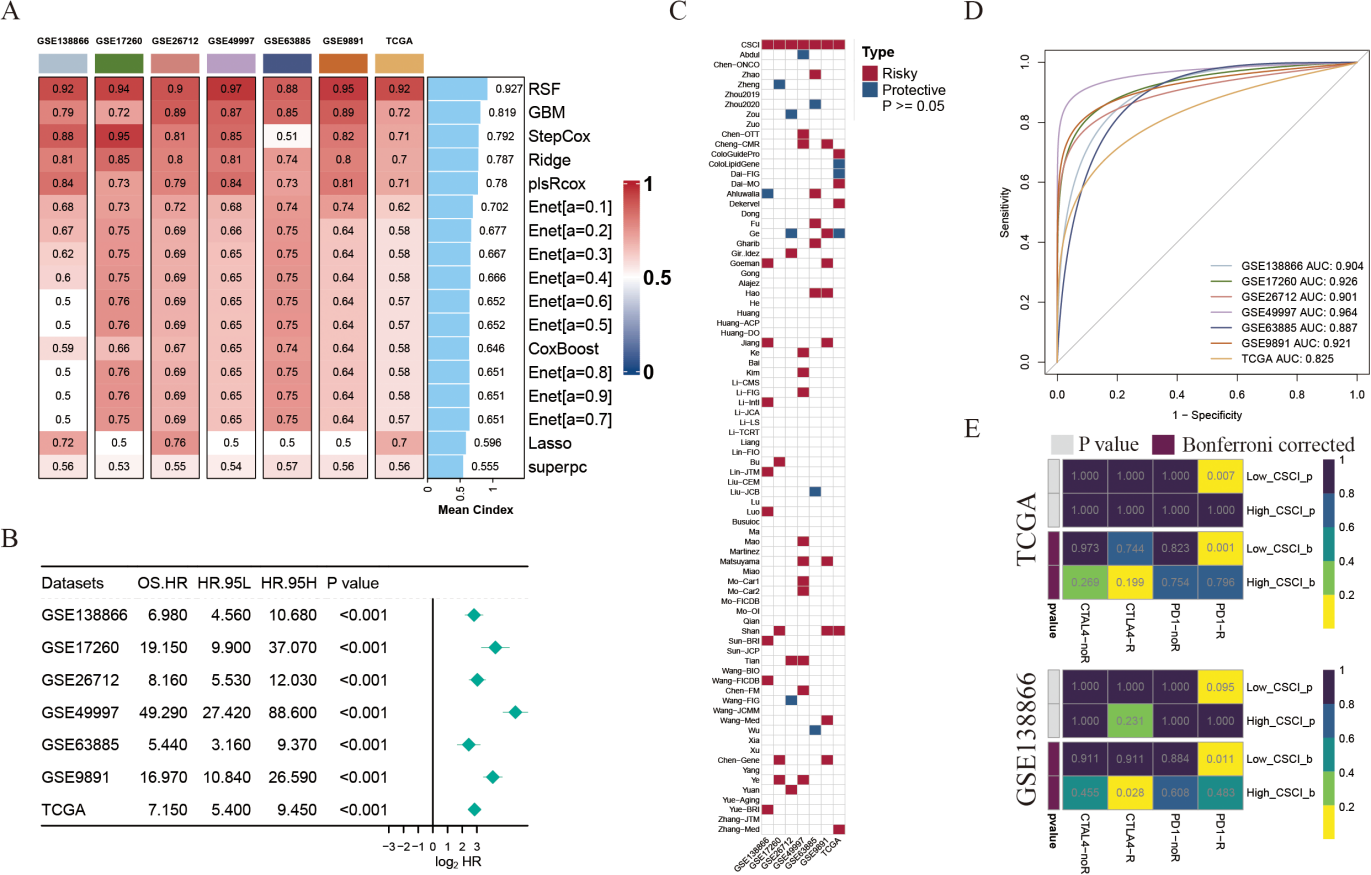
Construction of cancer stemness cell index based on large scRNA-seq and bulk RNA-seq cohorts. **(A)** The C-indexes of 9 algorithms in the 7 validation cohorts. **(B)** Univariate Cox regression of RSF score in seven OV cohorts. **(C)** Heatmap showing the stability of various 79 models compared with CSCI in 7 OV cohorts. Risky, worse survival rate (HR>1 & P<0.05); Protective, better survival rate (HR<1 & P<0.05). **(D)** ROC curves of CSCI for predicting clinical status in different OV cohorts. **(E)** Predicted response rate of different CSCI groups to immune checkpoint inhibitors (CTLA4 and PD1, R, Response; NR, no Response).

To further construct a prediction model for cancer stemness cell index (CSCI), we utilized nine machine learning algorithms on a combination of six GEO OV datasets and OV TCGA dataset. Subsequently, by employing these predictors, we computed the risk score for each sample in the seven cohorts. To assess the performance, we determined the average C-index for each algorithm. Intriguingly, the majority of these predictors demonstrated a notably high average C-index (**Figure 2A**). This observation can be partially attributed to the exceptional quality of our intrinsic cancer drivers and stemness markers. Out of all the models, random forest (RSF) exhibited the highest precision level (mean C-index > 0.90, **Figure 2A**) and was selected as the ultimate CSCI. Furthermore, using univariate cox analysis, we established a significant association between high CSCI in the seven cohorts and unfavorable survival outcomes (P<0.05, **Figure 2B**).

The advancement of next-generation sequencing and big data mining technologies has fostered the extensive exploration and development of gene expression-based signatures that can predict outcomes. To comprehensively compare the performance of the CSCI with other signatures, we systematically gathered published signatures from the past decade. In total, 79 signatures were included in this study, as detailed in **Supplementary Table S4**. It is worth noting that the robustness of the CSC index in terms of survival prediction surpassed that of almost all other models across seven cohorts, namely GSE138866, GSE17260, GSE26712, GSE49997, GSE63885, GSE9891, and TCGA, with mean AUC > 0.9 across the aforementioned cohorts (**Figures 2C, D**).

Considering the significance of cancer intrinsic driver and stemness mRNAs in forecasting the effectiveness of tumor immunotherapy, we employed the submap algorithm available on GenePattern websites to anticipate the probability of immune therapy response according to high- and low- CSCI clusters. Notably, an intriguing observation emerged whereby patients belonging to the low CSCI cluster manifested a substantial response towards PD-1 immunotherapy (P < 0.05, Bonferroni corrected P < 0.05, **Figure 2E**), thus exemplifying the exceptional forecasting capability of the CSCI model in the realm of PD-1 immunotherapy effectiveness.

### Cancer stemness cell index as a promising predictor for immunotherapy outcomes in pan-cancer cohorts

**Figure 3:**
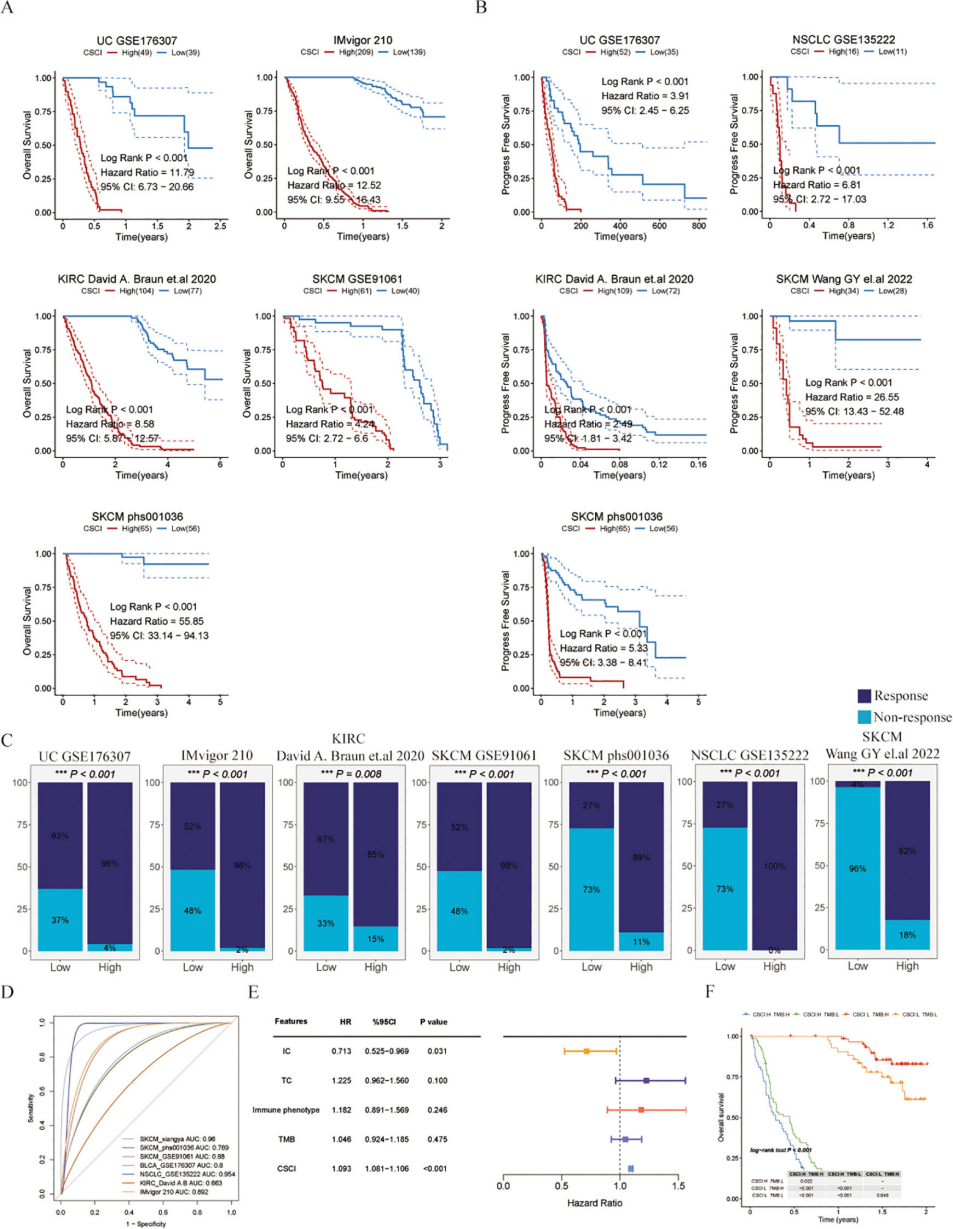
Cancer stemness cell index as a promising predictor for immunotherapy outcomes in pan-cancer cohorts. **(A)** Kaplan-Meier estimates of overall survival of patients treated with immunotherapy in UC (GSE176307), metastatic urothelial carcinoma (IMvigor210), KIRC (KIRC David **(A)** Braun et al., 2020), SKCM (GSE91061) and SKCM (phs000452.v3.p1). **(B)** Kaplan-Meier estimates of progression free survival of patients treated with immunotherapy in UC (GSE176307), NSCLC (GSE135222), KIRC (KIRC David **(A)** Braun et.al), SKCM (Wang GY el.al 2022) and SKCM (phs000452.v3.p1). **(C)**The response to immunotherapy in different CSCI group patients. Response means CR/PR and Non-response means PD/SD (CR, complete response; PD, progressive disease; PR, partly response; SD, stable disease). **(D)** ROC curves of CSCI for predicting response status in different ICI cohorts. **(E)** Multivariate Cox regression of CSCI and clinical features in metastatic urothelial carcinoma (IMvigor210). **(F)** Kaplan-Meier estimates of overall survival of patients with different groups in metastatic urothelial carcinoma (IMvigor210). The log-rank P value differences between each two groups are shown in the table.

In this study, we focused on evaluating the predictive value of the CSCI in immunotherapy outcomes using different datasets associated with PD-1/PD-L1 immune checkpoint inhibitors. Our findings consistently showed that patients with certain types of cancer (SKCM, UC, KIRC, or metastatic urothelial carcinoma) who had low CSCI scores had significantly improved overall survival (OS) or progression-free survival (PFS) after immunotherapy compared to those with high CSCI scores (**Figures 3A, B**), suggesting that high CSCI scores may compromise the benefits of immunotherapy. Furthermore, the response to PD-1/PD-L1 ICI treatment differed between patients with high and low CSCI scores. The pan-cancer analysis revealed that patients with elevated CSCI scores exhibited a poor response to ICI treatment, whereas over fifty percent of patients with low CSCI scores demonstrated a positive response (**Figure 3C**), including those with ovarian cancer (**Supplementary Figure S1I**). Patients with high CSCI scores responded poorly to treatment, while more than half of the patients with low CSCI scores responded, with a response rate of up to 96% (**Figure 3C**). Specifically, the high CSCI group primarily exhibited no response (progressive disease or stable disease), whereas the low CSCI group mostly showed a response (complete response or partial response). Importantly, our analysis demonstrated that CSCI had reliable predictive value in predicting PD-1/PD-L1 ICI immunotherapy response, as indicated by the area under the curve (AUC) values. The AUC curve showed strong predictive performance, with a mean AUC > 0.8 in the seven cohorts analyzed (**Figure 3D**). Additionally, we conducted further analysis using the IMvigor210 dataset and found that even after removing samples with incomplete clinical information, CSCI remained an independent predictor of immunotherapy outcomes. Notably, it was even more significant than PD-L1 expression in tumor cells (TC), immune phenotype, or tumor mutation burden (TMB), based on multivariate Cox regression analysis (**Figure 3E**). To extend the clinical utility of our model, we investigated the combination of CSCI with other commonly used immunotherapy response markers. We specifically examined the combination of CSCI with TMB, a well-established predictor of immunotherapy efficacy. Our results showed that patients with low CSCI scores and high TMB had the most optimal immunotherapy outcomes, whereas patients with high CSCI scores had the poorest benefits from immunotherapy (**Figure 3F**).

In summary, our study provides valuable insights into the predictive value of CSCI in immunotherapy outcomes. High CSCI scores may compromise the benefits of immunotherapy, while low CSCI scores are associated with improved survival and treatment response. The combination of CSCI with other biomarkers, such as TMB, may further enhance the stratification of patients for immunotherapy.

### Cancer intrinsic drivers and stemness properties to portrait the path of OV malignant cells progression

The concept of cancer stemness pertains to the tumors’ capacity for self-renewal and differentiation into various lineages, often observed in the initial phases of tumor differentiation. Consequently, we postulate that the evaluation of tumor stemness and intrinsic driver genes enables the quantification of tumor proliferation and progression in ovarian cancer.

**Figure 4:**
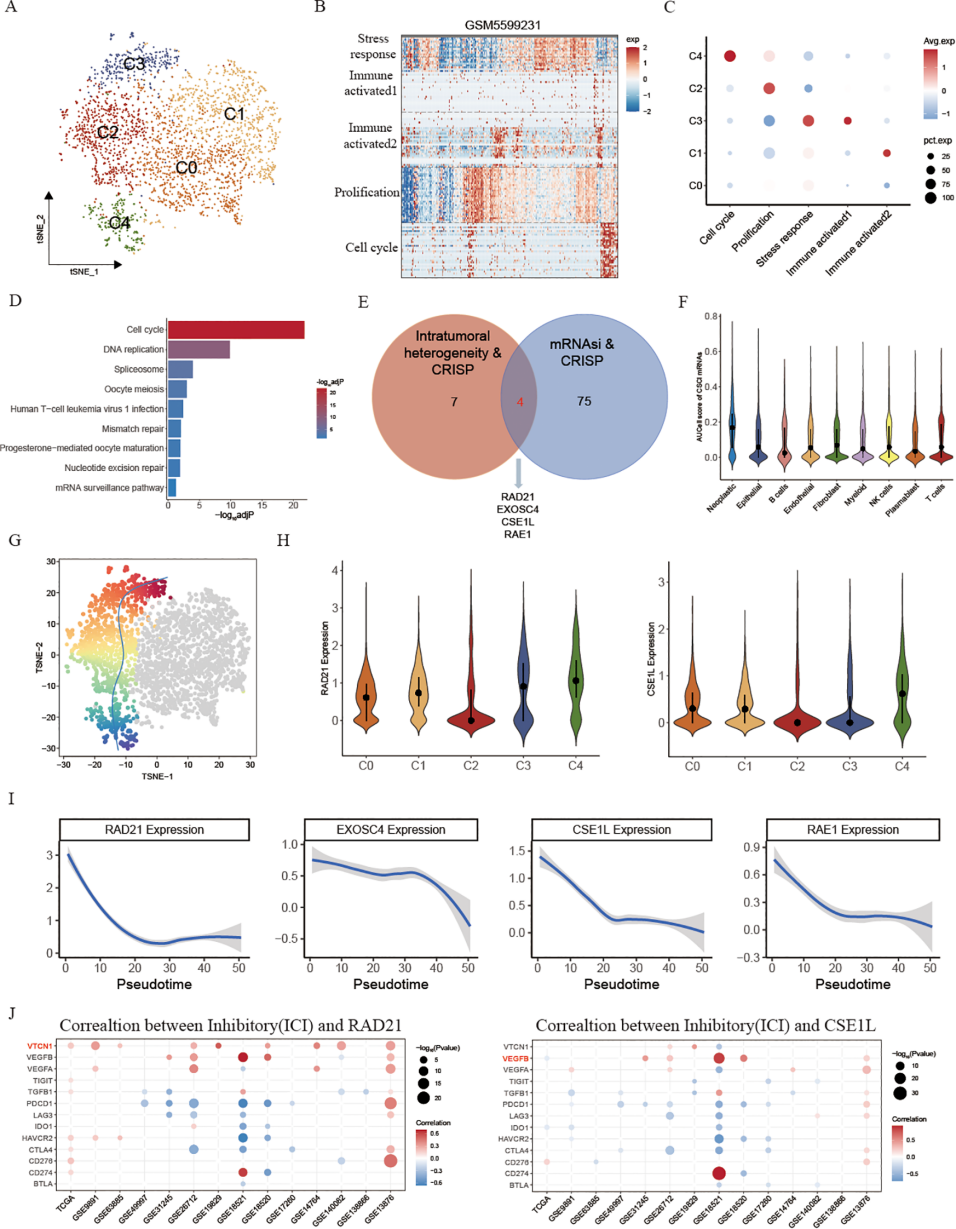
Cancer intrinsic drivers and stemness properties to portrait the path of OV progression. **(A)** tSNE plot showing the composition of 5 main subtypes derived from OV malignant cells. **(B)** Heatmap showing expression programs derived in a representative patient using NMF. **(C)** Relative expression scores of meta-programs in each OV malignant cell cluster. **(D)** KEGG enrichment of cancer intrinsic driver and stemness associated mRNAs. **(E)** Venn diagram showing the overlap between intratumoral heterogeneity driven genes and cancer stemness associated genes. **(F)** AUCell score of selected 4 intrinsic variation driven cancer stemness associated genes in different cell types. **(G)** t-SNE visualizes the evolutionary trajectory of tumor cells calculated by slightshot. **(H)** Violin plots illustrating the expression of RAD21 and CSE1L across different malignant cell subtypes. **(I)** The relationship between gene expression and evolutionary time. **(J)** The correlation between inhibitory and RAD21, CSE1L in fifteen OV datasets.

In order to evaluate this hypothesis, our initial step involved reorganizing OV neoplastic cells obtained from scRNA-seq. This process resulted in the discovery of 5 distinct neoplastic subclusters (**Figure 4A**). Next, to analyze the diversity in gene expression among the tumor cell population, we took a step further and started identifying intra-tumor expression programs. These programs included groups of genes that were found to be co-expressed in each tumor sample. This was accomplished by employing a technique called non-negative matrix factorization (NMF). The resulting expression programs served as gene modules, indicating high expression levels in specific subsets of tumor cells within each individual tumor. A representative tumor, GSM5599631, was used to showcase the NMF outcome (**Figure 4B**). Overall, we analyzed a total of 31 intra-tumor expression programs across seven OV tissues, and further categorized them into five shared meta-programs (MPs) found in multiple tumors (**Figure 4C**; **Supplementary Figure S2B**; **Supplementary Tables S5, S6**). Module1 (Stress response) was characterized by expression of genes such as FOS and JUN, thus representing a stress-response-related signature in tumor cells. Module2 (Immune activated) was characterized by enrichment of allograft rejection. Additionally, we found that Module3 (Immune activated) was enriched for Interferon gamma/alpha response, which are related to the immune activated pathways. Module4 (Prolification) was characterized by enrichment of cancer prolification pathways, including myc targets V1 and mtorc1 signaling. Module5 (Cell cycle) was characterized by expression of Cell cycle-related genes such as MKI67 and CDKN3 and enrichment of cell cycle, E2F targets and G2M checkpoint pathways (**Supplementary Table S5**), which was considered to be the inception of tumor development. Interestingly, we found cancer intrinsic driver and stemness associated mRNAs were significantly enriched for cell cycle and DNA replication pathways (**Figure 4D**), implying the association between tumor stemness-related genes and the initiation of tumorigenesis. Then, we performed an overlap analysis of the gene set comprising tumor intrinsic driver genes impacting tumor progression and tumor stemness-related genes (**Figure 4E**). This enabled us to identify the core genes associated with tumor stemness induced by tumor intrinsic variations and finally we selected four genes: RAD21, EXOSC4, CSE1L and RAE1. Earlier investigations demonstrated that the amplification of RAD21 epigenetically restrains interferon signaling, thus promoting immune escape in ovarian cancer and having the potential to function as a molecular sign for immunotherapy regarding ovarian cancer (11). Using AUCell, we found that these four genes exhibit significantly higher predictive efficacy for malignant tumor cells compared to all other tumor intrinsic driver and stemness-related genes (**Figure 4F**, **S2A**). Next, pseudo-time analysis was performed on the 5 malignant subclusters (**Figure 4G**), and cell cycle cluster C4 was serves as the initiation of tumorigenesis, followed by evolutionary progression along the proliferative C2 cluster. Consistent with this, we found that RAD21 was highest expressed in the C4 subclass, and interestingly, we also found that CSE1L was also in line with this trend, whereas there were no significant differences observed in EXOSC4 and RAE1 among various subgroups (**Figures 4H**, **S2C**). Moreover, we also found EXOSC4, CSE1L, and RAE1 were high expressed in no-response OV patients, further proving that stemness-related genes may affect the effect of immunotherapy (**Supplementary Figure S2D**). The evolutionary trajectory also verified this result, suggesting its utility in predicting OV malignant cell progression (**Figure 4I**). Immune suppressive factors are the main obstacles influencing tumor immunotherapy (31). Therefore, to investigate the mechanisms by which RAD21 and CSE1L affect the efficacy of immunotherapy, we examined the correlations between various immune suppressive factors and RAD21/CSE1L in 14 ovarian cancer datasets (**Figure 4J**). Our findings indicated that RAD21 primarily activates the expression of VTCN1 (B7-H4), while CSE1L is significantly associated with VEGFB. Prior investigations have shown that the co-inhibitory molecule B7-H4 negatively controls the T cell immune response and helps with immune escape through suppressing the proliferation rate, cytokine secretion, and cell cycle progression of T cells (32). Hence, we suspect that the amplification of RAD21 promotes immune escape by activating B7-H4 expression in OV malignant cells, whereas CSE1L potentially influences tumor progression by stimulating the expression of VEGF.

Collectively, these findings suggested that stemness originating from cancer’s intrinsic variability could function as predictive markers for the progression of ovarian cancer. Additionally, RAD21 and CSE1L possess the capability to be employed as immunotherapy predictions.

### Investigating CSE1L-related immune landscapes and providing drug therapy strategies

To verify the performance of cancer intrinsic variation driven stemness in clinical implementation, we further investigated the intrinsic drivers and stemness properties applied in CSCI. Among these drivers, RAD21 and CSE1L were found to be tumor intrinsic copy number variation-driven factors associated with tumor stemness and tumor evolution. Previous studies have shown that RAD21 amplification epigenetically interacts with YAP/TEAD4 transcriptional co-repressors, recruiting the NuRD complex to inhibit interferon (IFN) signaling and promote immune evasion in ovarian cancer (11). However, limited research has focused on the role of CSE1L in the immunotherapy response of OV cancer.

**Figure 5:**
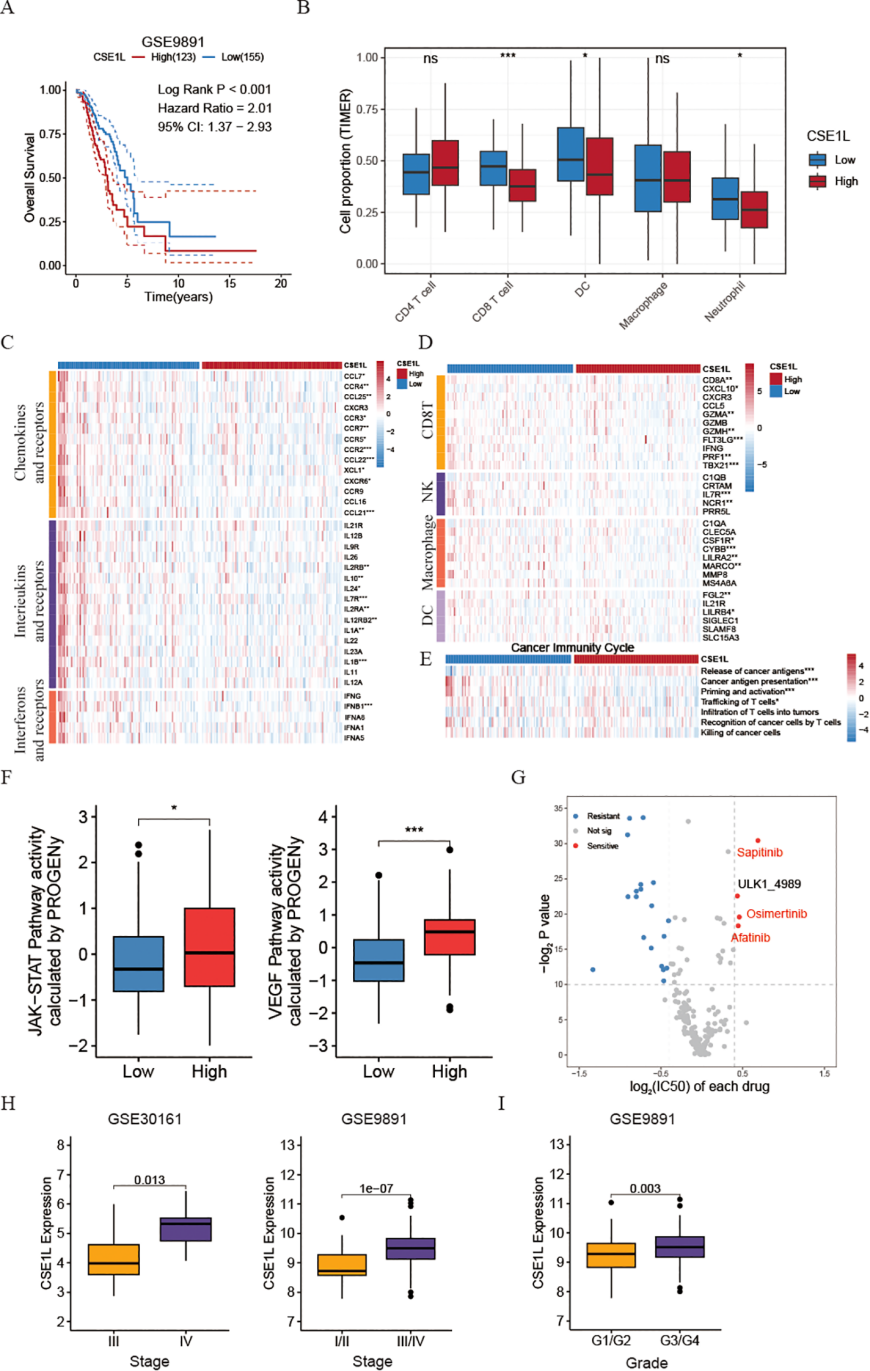
Investigating CSE1L-related immune landscapes and providing drug therapy strategies. **(A)** Kaplan-Meier estimates of overall survival of patients in OV dataset (GSE9891). **(B)** Box plots comparing the proportion of immune cells between low- and high- CSE1L groups (GSE26712). **(C)** Heatmap of the expression of chemokines and their corresponding receptors, interferons and their corresponding receptors, and interleukins and their receptors in low- and high-CSE1L groups (GSE26712). **(D)** Heatmap of the GSVA score of seven steps in cancer immunity cycle in low- and high- CSE1L groups (GSE26712). **(E)** Heatmap of the expression of effector markers of tumor-infiltrating immune cells in low- and high-CSE1L groups (GSE26712). **(F)** Box plots comparing the activity of JAK/STAT and VEGF pathway between low- and high- CSE1L groups (GSE26712). **(G)** Dot plot showing the drug drug susceptibility of chemotherapy drugs. The x-axis represents the log2FC of IC50, where a positive value indicates that the IC50 of the low CSE1L group is greater than that of the high CSE1L group. The y-axis represents -log2 P-value (GSE26712). The drugs highlighted in red are chemotherapy drugs that inhibit VEGF. **(H)** Box plots comparing the expression of CSE1L between low- and high- Stage groups in GSE30161 and GSE9891. **(I)** Box plots comparing the expression of CSE1L between low- and high- Grade groups in GSE9891. (Wilcoxon test; ns, no significant; *P < 0.05; ***P < 0.001).

Kaplan-Meier survival analysis revealed a significant association between CSE1L and poor prognosis in ovarian cancer (**Figure 5A**). To further understand the function of CSE1L in immunotherapy, we assessed immune cell infiltration in high- and low- CSE1L group patients in a GEO cohort. Using TIMER web, they found that most immune cells, such as CD8+ T cells, DC cells, and Neutrophil cells, were significantly higher in the low-CSE1L group compared to the high-CSE1L group (**Figure 5B**). This suggests that CSE1L is capable of suppressing the activation of immune cells, potentially inhibiting the effectiveness of immunotherapy.

Tertiary lymphoid structures (TLS) function as germinal centers for immune cells within the tumor microenvironment (TME), therefore, we assessed the expression of different chemokines involved in TLS formation. Interestingly, a significant upregulation of most chemokines was observed in the low- CSE1L group. Specifically, patients with low CSE1L levels displayed increased levels of CCL7, CCR4, CCL25, CXCR3, CCR3, CCR7, CCR5, CCR2, CCL22, CCL21, and XCL1 (**Figure 5C**). In brief, the expression of these chemokines, crucial for attracting immune cells to the TME, was comparatively lower in the high CSE1L group when compared to the low CSE1L group. Additionally, we noted that the lower expression of CSE1L was linked to several interferons and their respective receptors (e.g., IFNB1), as well as the majority of interleukins and their receptors (**Figure 5C**). Similarly, CSE1L demonstrated a negative correlation with the effector genes of these immune cells infiltrating the tumor (**Figure 5D**).

The functions of the chemokine system and other immunomodulators directly manifest as the activities of the cancer immunity cycle (33). Triggering the release of cancer antigens (step1) occurs in the high-CSE1L group, suggesting that amplification of CSE1L leads to the emergence of OV malignant cells. This amplification enables the immune system to recognize cancer-released antigens during the initiation stage of tumorigenesis. As the tumor continues to evolve, the expression of CSE1L decreases, resulting in a reduction in tumor cell stemness. Subsequently, other cycles, including cancer antigen presentation (step2), priming and activation (step3), and trafficking of T cells (step4), are activated (**Figure 5E**).

Moreover, to investigate the potential mechanism behind the effectiveness of CSE1L in tumor progression, we performed an assessment of the activity of 14 signaling pathways associated with cancer using the R package “PROGENy”. Through the implementation of the Kruskal-Wallis test, our analysis revealed that the JAK-STAT and VEGF pathways were specifically activated in patients belonging to the high CSE1L group (**Figure 5F**), suggesting that CSE1L plays a crucial role in activating the JAK-STAT signaling pathway and subsequently suppressing immune system activation. Additionally, it possesses the ability to foster angiogenesis by stimulating the VEGF signaling pathway. Then, to validate this founding, we stratified malignant tumor cells based on the expression of CSE1L into CSE1L+ malignant cells and CSE1L- malignant cells. Through GSEA, we discovered a significant activation of the VEGF signaling pathway in cells expressing CSE1L, further confirming our findings (**Figure 5G** and **Supplementary Figure S2E**). To further validate the role of CSE1L in ovarian cancer, we extended our investigation to include two additional datasets, GSE30161 and GSE9891. Intriguingly, we observed that the expression of CSE1L increased with the progression of tumor malignancy (**Figures 5H, I**).

Considering the crucial role of combining chemotherapy and immunotherapy in treating OV patients, we additionally examined the variation in chemotherapy response between high- and low- CSE1L groups. Intriguingly, using oncoPredict R package, our findings revealed that patients belonging to the high-CSE1L group exhibited heightened sensitivity towards VEGF inhibitor medications such as Sapitinib, Osimertinib, and Afatinib (**Figure 5G**). To further verify this result, we then also calculated the IC50 of Sorafenib, a VEGF inhibitor, using pRRopetic R package. We found that the IC50 of Sorafenib was also higher in low CSE1L group than that in high CSE1L group (**Supplementary Figure S2F**).

In summary, the CSE1L has the potential to function as a dependable predictor of patients’ prognosis regarding immunotherapy. Additionally, it can serve as an efficient biomarker in foreseeing patients’ responsiveness to chemotherapeutic medications.

### CSE1Lpromotes progression of ovarian cancer *in vitro*

**Figure 6:**
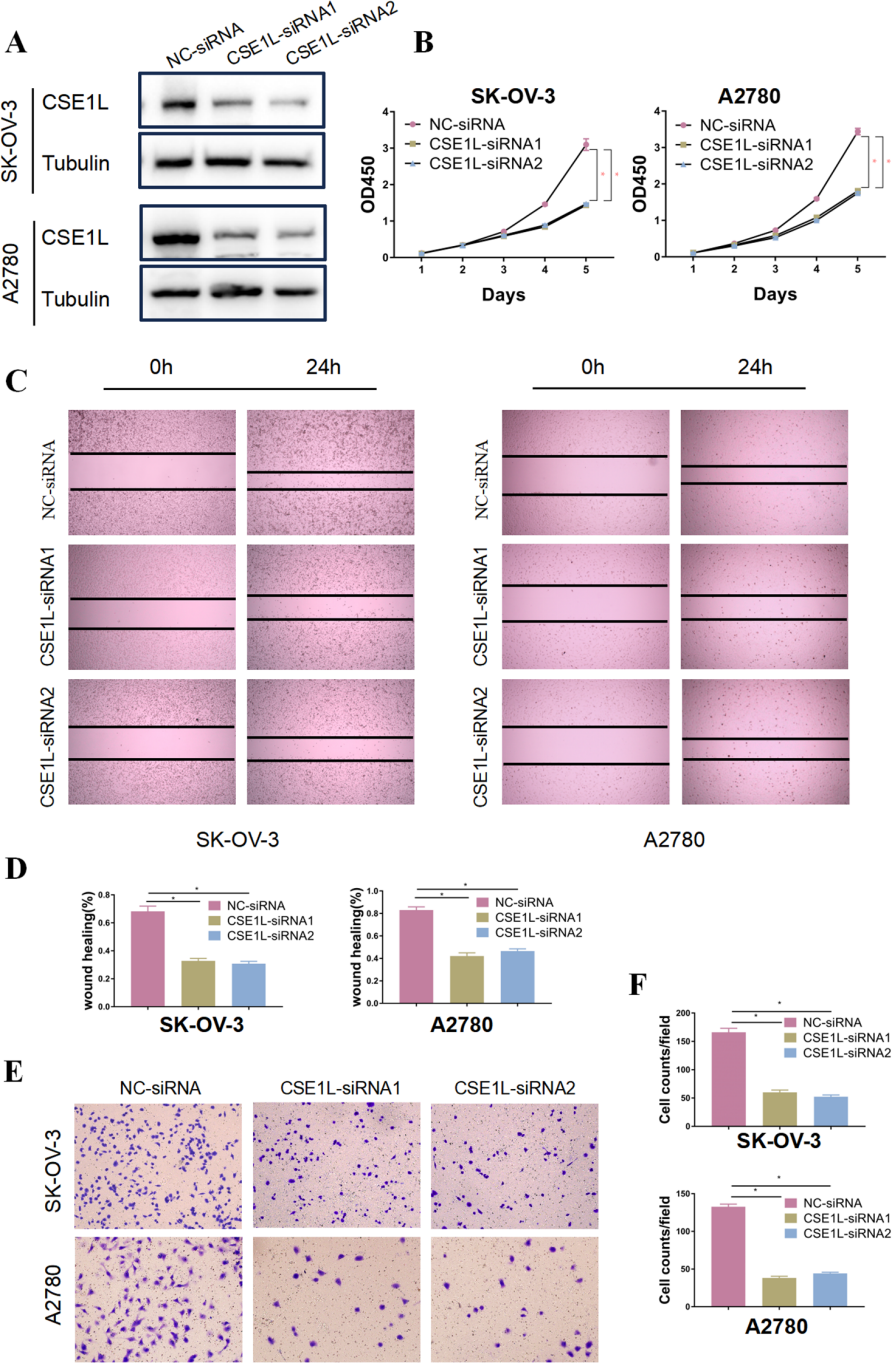
CSE1L promotes progression of ovarian cancer *in vitro*. **(A)** Representative immunohistochemistry results showed that CSE1L expression was significantly upregulated in tumor tissues compared to adjacent non-cancerous tissues. **(B)** Validation of CSE1L of knockout in OVCAR-3 and A2780 cell lines by Western Blotting analysis. **(C)** Proliferation ability of CSE1L-knockout ovarian cancer cells detected by CCK8 assay. **(D, E)** Evaluation and quantitative analysis of the migration ability of CSE1L-knockout OVCAR-3 and A2780 cell lines using wound healing assays. **(F)** Analysis and quantitative assessment of the effect of CSE1L knockdown on the migration ability of ovarian cancer cells using Transwell assays. Scale bar, 100μm. (Wilcoxon test; *P < 0.05).

To further verify the oncogenic role of CSE1L in ovarian cancer, Immunohistochemistry was conducted, and the results indicated that CSE1L expression was significantly upregulated in tumor tissues compared to adjacent non-cancerous tissues (**Figure 6A**). Also, CSE1L was knocked down in SK-OV-3 and A2780 cell lines, and the effectiveness of the knockdown was confirmed by Western blot analysis at the protein level (**Figure 6B**). Notably, the knockdown of CSE1L significantly inhibited cell proliferation in the SK-OV-3 and A2780 cell lines (**Figure 6C**), emphasizing the crucial role of CSE1L in promoting ovarian cancer cell growth. Additionally, in the scratch wound healing assay, knocking down CSE1L significantly impaired the migration ability of SK-OV-3 and A2780 cell lines (**Figure 6D**). This was further confirmed by Transwell assays, demonstrating the important function of CSE1L in promoting ovarian cancer cell migration (**Figures 6E, F**).

## Discussion

Tumor immune evasion and the effectiveness of immunotherapy are influenced by tumor intrinsic heterogeneity and stemness, which are crucial elements. While the relationship between cancer stemness and anti-tumor immunity has been extensively investigated (6, 8), there is currently no reported direct evidence uncovering the connection between tumor stemness and ICI response in OV. Additionally, prior studies have neglected to recognize the predictive capacity of tumor stemness in determining the response to ICI in OV (34).

In this study, we initially detected inherent copy number variations (CNVs) that promote malignancy in ovarian cancer cells at the single-cell level. These CNVs encompass gains of chromosomal 20q and 8q, as well as loss of chromosomal 5q. Subsequently, through the integration of multiple omics data, including single-cell, transcriptome, and CRISPR cell line data, and utilizing a previously published stemness index (9), we identified genes associated with tumor stemness, which are also influenced by intrinsic variations in tumors. It is worth noting that we found a reverse relationship between stemness and the outcomes of ICIs, which was supported by the findings from a scRNA-Seq cohort of SKCM (10). Following this, to evaluate the prognostic impact of tumor stemness genes in ovarian cancer patients, we utilized various machine learning algorithms to construct a predictive model for CSCI. The performance of this model was then validated across seven independent cohorts using multiple evaluation metrics. Ultimately, the Random Survival Forest (RSF) model emerged as the selected CSCI, as it demonstrated significantly enhanced stability and accuracy compared to previously established models. Furthermore, our investigations unveiled the effectiveness of the CSCI in predicting the therapeutic efficacy of PD-1 immune checkpoint inhibitors. Cancer Stem Cells have been identified in nearly all types of solid tumors (6). Based on these findings, we postulated that the CSCI could have broad applicability in predicting the response to immunotherapy across diverse cancer types. Consequently, we conducted a large-scale comprehensive analysis to assess the accuracy of the CSCI in predicting the response to immunotherapy in other cancer types. Remarkably, the CSCI exhibited excellent performance in predicting ICI response across multiple independent cohorts utilizing bulk RNA-Seq data, with a mean Area Under the Curve (AUC) exceeding 0.8.

CSCs are self-renewal cells that promote tumor initiation, progression, and metastasis (6). In comparison to regular tumor cells, tumor stemness exert their influence primarily during the initial stages of tumor development. Through single-cell data analysis, we have discovered a subset of tumor cells positioned at the initial stage of tumor development. These cells exhibit specific activation of pathways such as the cell cycle and undergo evolution towards highly proliferative tumor cells. Consistent with these findings, our KEGG enrichment analysis of CSC gene set also reveals significant enrichment in the cell cycle pathway. Previous investigations have demonstrated that the improper activation of the cell cycle signaling pathway results in a boost in the expression levels of transcription factors (for example, CDK, MKI67, and p53), potentially facilitating the formation and sustenance of tumor stemness (35). This indicates that the CSC gene set can depict the evolutionary trajectory of malignant ovarian tumor cells. Furthermore, we have identified two endogenously amplified core genes, RAD21 and CSE1L, promoting the progress of OV malignant cells. These two genes serve as potent tools for delineating the trajectory of malignant cell progression in OV. Deng P et al. discovered that RAD21, an essential adhesive protein, its amplification promotes interaction with YAP/TEAD4 and recruits NuRD to form a complex that suppresses IFN signaling pathway gene expression, thereby inhibiting the response to immunotherapy (11). Moreover, we have also observed that RAD21 facilitates the upregulation of VTCN1 (B7-H4) expression. B7-H4, as an immune checkpoint molecule expressed on antigen-presenting cells, functions to interact with CD8+ T cells, thereby suppressing the activity of immune cells and inhibiting their anti-tumor response (36). Given the paucity of research on the role of CSE1L in ovarian cancer, we also investigated its impact on immunotherapy and provided a therapeutic strategy involving chemotherapy drugs. We found that CSE1L exhibited a significant association with immune cell suppression, which was further validated through the use of immune factors and markers of immune cells. Additionally, we discovered that CSE1L not only activates the VEGF signaling pathway but also triggers the JAK/STAT signaling pathway. This suggests that CSE1L may promote OV tumor progression through the activation of the JAK/STAT signaling pathway (37, 38). Finally, the IC50 values of drugs demonstrated that the high-CSE1L group displayed heightened sensitivity to VEGF inhibitor drugs, including Sapitinib, Osimertinib, and Afatinib. Collectively, these results implicate that cancer intrinsic variation derived stemness could serve as predictive indicators of disease progression in ovarian cancer. Moreover, RAD21 and CSE1L have the potential to be utilized as novel predictive targets for immunotherapy.

Although the notable precision of the CSCI in forecasting the effectiveness of immunotherapy should be emphasized, it is crucial to recognize specific constraints within this investigation. Initially, the OV model’s capacity to predict ovarian cancer immunotherapy outcomes relies on projections derived from the submap algorithm, and the accuracy of the CSCI necessitates confirmation with actual OV ovarian cancer immunotherapy cohorts. Additionally, our analysis could not definitively assess whether essential genes in ovarian cancer or stemness-related genes contributed more substantially to the model’s predictive power. Traditional linear models, such as LASSO or Cox regression, provide interpretable feature weights, where each gene’s importance is reflected in its coefficient. In contrast, random forest evaluates feature importance through alternative metrics (e.g., Gini impurity), which do not yield directly comparable weights between gene categories. As a result, we were unable to draw conclusive comparisons regarding the relative importance of these two gene sets in our model. Furthermore, additional experimental verification is imperative to unveil the molecular mechanisms underlying the relationship between CSE1L, immunotherapy response, and tumor advancement.

## Conclusion

To summarize, a stable and reliable CSC signature was developed through integrative analysis of CRISPR OV cell lines, as well as large-scale bulk OV tissues and single cell cohorts. This signature enables the stratification of OV patients and the prediction of immunotherapy outcomes. Our study is the pioneering investigation that comprehensively examines the correlation between cancer stemness and immunotherapy in OV. It offers a broad foundation for comprehending the significance of cancer stemness in immuno-oncology, clinical advantages, and practical applications. In light of our findings, this research expands our understanding of the relationship between cancer stemness and immunotherapy in OV, opening up new possibilities for treatment approaches.

## Data Availability

The datasets presented in this study can be found in online repositories. The names of the repository/repositories and accession number(s) can be found in the article/**Supplementary Material**.

## Supplementary material

Supplementary Figure 1 Investigating intrinsic heterogeneity in OV through single-cell analysis. **(A)** tSNE plot showing the composition of eight main cell types derived from OV tissues. **(B)** Heatmap showing expression of each cell markers in each cell type. **(C)** Heatmap showing copy number variation of reference cells and epithelial cells. **(D)** Heatmap showing copy number variation of reference cells and malignant epithelial cells. **(E)** Evolutionary phylogenetic tree of malignant epithelial cells. **(F)** UpSet plots revealed the numbers of genes located on chromosome 5 shared by the subclones with 5q loss gain in OV malignant cells. **(G)** UpSet plots revealed the numbers of genes located on chromosome 8 shared by the subclones with 8q gain gain in OV malignant cells. **(H)** UpSet plots revealed the numbers of genes located on chromosome 20 shared by the subclones with 20q gain gain in OV malignant cells. **(I)** The response to immunotherapy in different CSCI group OV patients in GSE188249.

Supplementary Figure 2 OV malignant cell expression programs. **(A)** Heatmap depicting shared expression meta-programs across all patients. Module1: GSM5599225_1, GSM5599226_6 and GSM5599231_4; Module2: GSM5599225_5, GSM5599228_2; Module3: GSM5599228_3, GSM5599230_1; Module4: GSM5599226_2, GSM5599225_4, GSM5599231_2; Module5: GSM5599229_1, GSM5599226_1, GSM5599228_1, GSM5599225_3, GSM5599231_1. **(B)** AUCell score of total intrinsic variation drivers and cancer stemness associated genes in different cell types. **(C)** Violin plots illustrating the expression of EXOSC4 and RAE1 across different malignant cell subtypes. **(D)** Box plots comparing the expression of RAD21, EXOSC4, CSE1L, and RAE1 between immunotherapy response (R) and no-response (NR) groups in OV patients (GSE188249). **(E)** GSEA enrichment of VEGF pathway between GSE1L+ malignant and CSE1L- malignant cells. **(F)** Box plots comparing the IC50 of Sorafenib between low- and high- CSE1L groups in GSE26712.
