# Supplementary figures and images for: Multi-omics analysis and experiments uncover the link between cancer intrinsic drivers, stemness, and immunotherapy in ovarian cancer with validation in a pan-cancer census
Source: Front Immunol. 2025 May 8;16:1549656. doi: 10.3389/fimmu.2025.1549656 (PMC12095155; doi:10.3389/fimmu.2025.1549656)

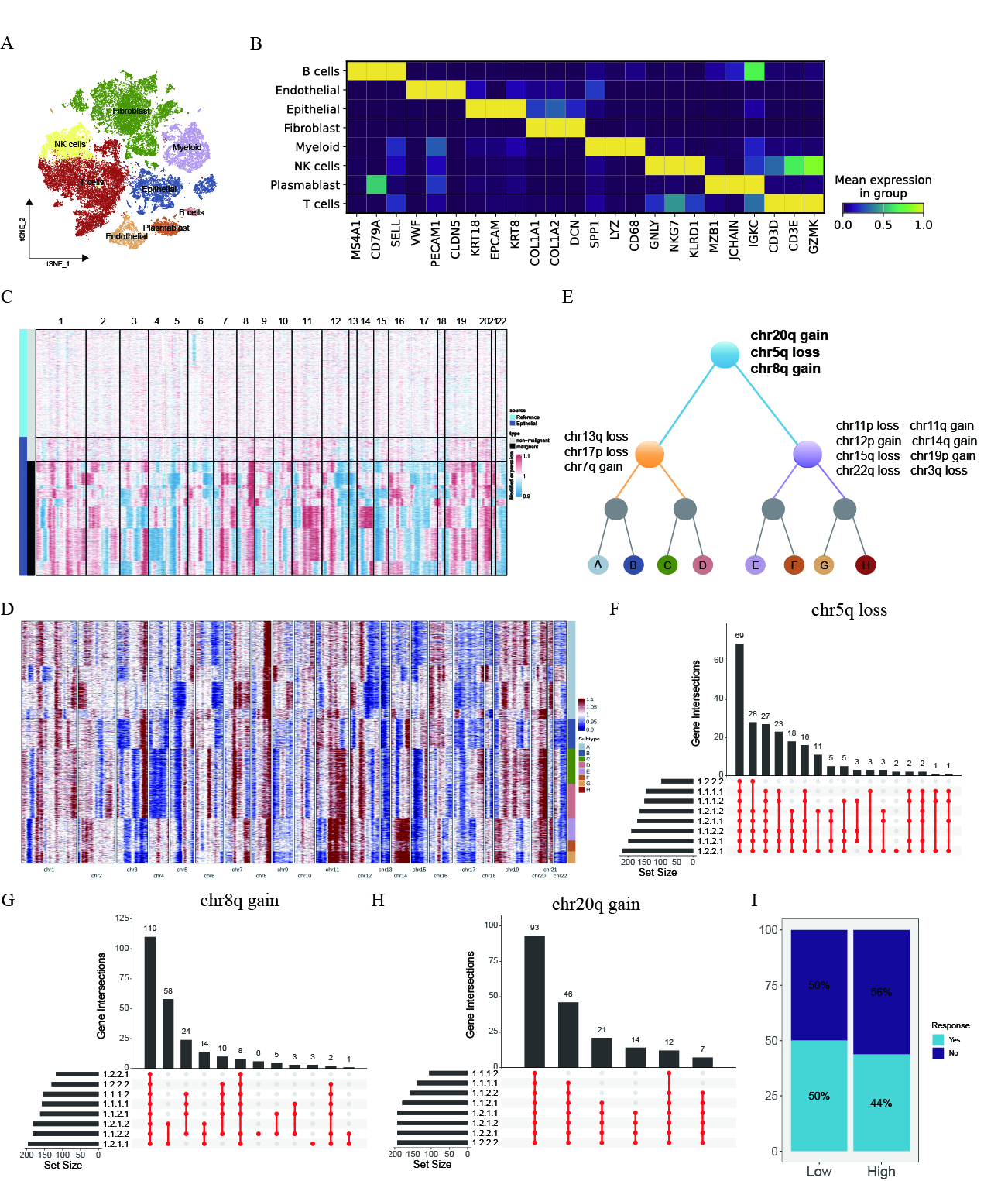

Supplement: Supplementary Figure 1 — Investigating intrinsic heterogeneity in OV through single-cell analysis. (A) tSNE plot showing the composition of eight main cell types derived from OV tissues. (B) Heatmap showing expression of each cell markers in each cell type. (C) Heatmap showing copy number variation of reference cells and epithelial cells. (D) Heatmap showing copy number variation of reference cells and malignant epithelial cells. (E) Evolutionary phylogenetic tree of malignant epithelial cells. (F) UpSet plots revealed the numbers of genes located on chromosome 5 shared by the subclones with 5q loss gain in OV malignant cells. (G) UpSet plots revealed the numbers of genes located on chromosome 8 shared by the subclones with 8q gain gain in OV malignant cells. (H) UpSet plots revealed the numbers of genes located on chromosome 20 shared by the subclones with 20q gain gain in OV malignant cells. (I) The response to immunotherapy in different CSCI group OV patients in GSE188249. [file Image1.tif]

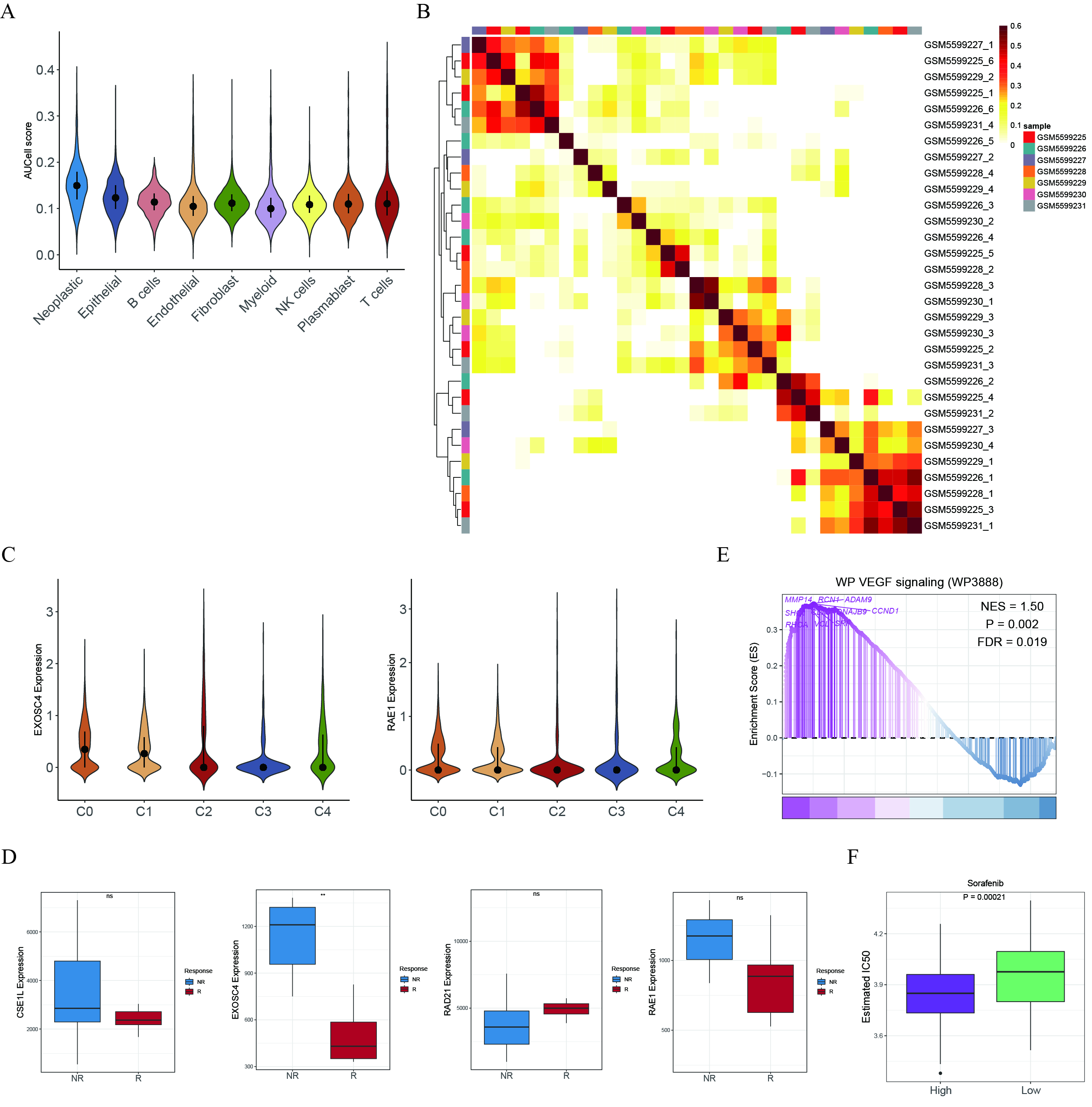

Supplement: Supplementary Figure 2 — OV malignant cell expression programs. (A) Heatmap depicting shared expression meta-programs across all patients. Module1: GSM5599225_1, GSM5599226_6 and GSM5599231_4; Module2: GSM5599225_5, GSM5599228_2; Module3: GSM5599228_3, GSM5599230_1; Module4: GSM5599226_2, GSM5599225_4, GSM5599231_2; Module5: GSM5599229_1, GSM5599226_1, GSM5599228_1, GSM5599225_3, GSM5599231_1. (B) AUCell score of total intrinsic variation drivers and cancer stemness associated genes in different cell types. (C) Violin plots illustrating the expression of EXOSC4 and RAE1 across different malignant cell subtypes. (D) Box plots comparing the expression of RAD21, EXOSC4, CSE1L, and RAE1 between immunotherapy response (R) and no-response (NR) groups in OV patients (GSE188249). (E) GSEA enrichment of VEGF pathway between GSE1L+ malignant and CSE1L- malignant cells. (F) Box plots comparing the IC50 of Sorafenib between low- and high- CSE1L groups in GSE26712. [file Image2.tif]

## Supplementary Information

Full-length Western-blots of Figure 6A

SK-OV-3

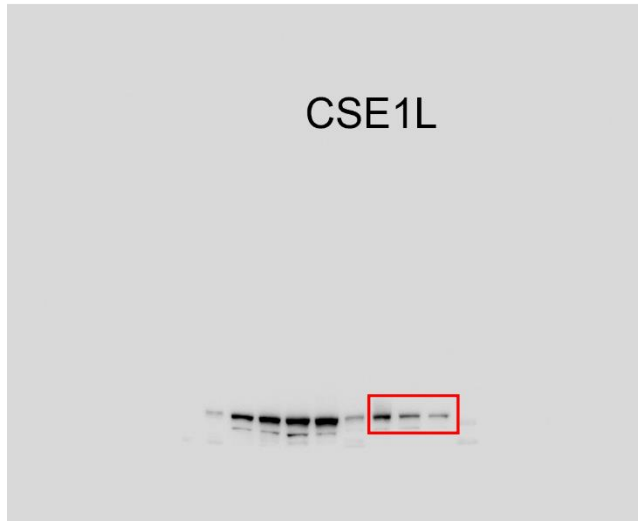

A2780

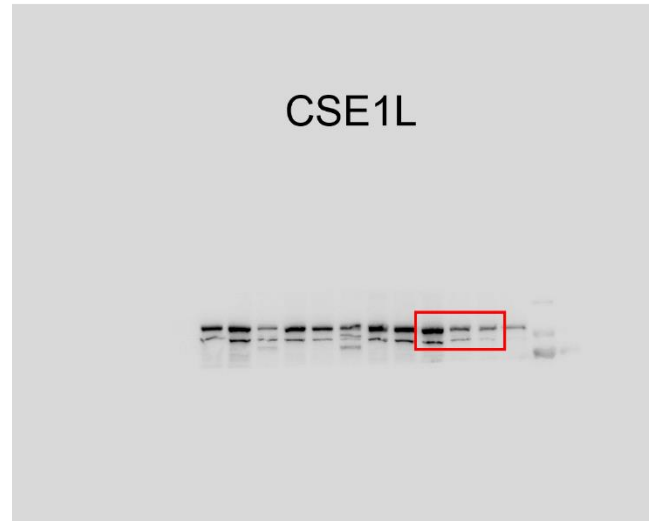

Tubulin

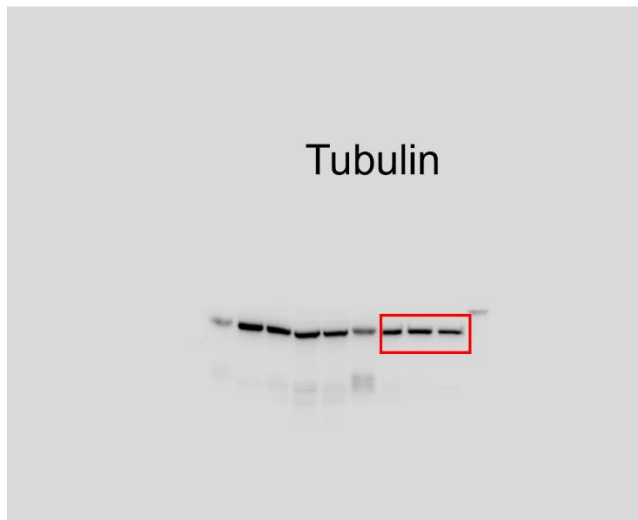

Tubulin

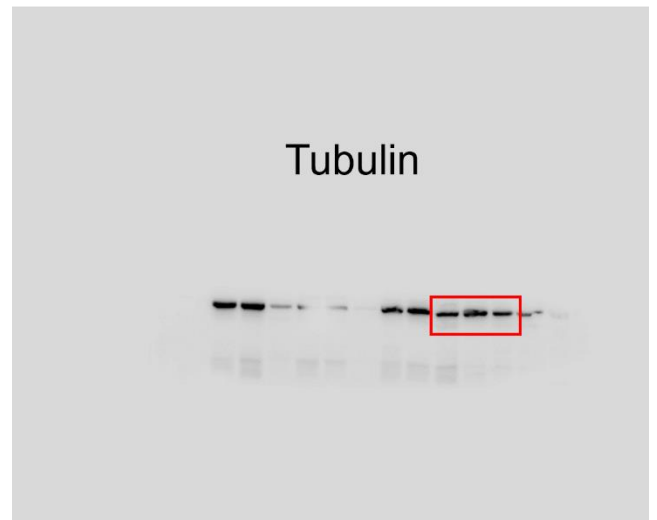

Supplement: Supplementary file 4 [file DataSheet2.pdf]
