# Supplementary material for: Multi-omics analysis and experiments uncover the link between cancer intrinsic drivers, stemness, and immunotherapy in ovarian cancer with validation in a pan-cancer census
Source: Front Immunol. 2025 May 8;16:1549656. doi: 10.3389/fimmu.2025.1549656 (PMC12095155; doi:10.3389/fimmu.2025.1549656)
Supplement: Supplementary file 3 [file DataSheet1.pdf]

English translation version of ethics approval

Scientific Research Ethics Committee of Binzhou Medical University Hospital

Ethical approval for clinical research projects

【2024】No. KYLL-202

**Study Title**	Multi omics analysis and molecular marker study of immunotherapy for ovarian cancer
**Institution**	Binzhou Medical University Hospital
**Research Leader**	Lei Han
**Ethical review opinions**	After review by our ethics committee, it is deemed that the scope of research content and research methods involved in this study meet the relevant requirements of medical ethics, and it is agreed to this study.
**Name of Ethics Committee**	Scientific Research Ethics Committee of Binzhou Medical University Hospital (official seal)
**Signature of chairman**	Xiaomin Zhang
**Approval date**	
**Tel**	0543-3258355
